# Readiness to tackle chronicity in Spanish health care organisations: a two-year experience with the Instrumento de Evaluación de Modelos de Atención ante la Cronicidad/Assessment of Readiness for Chronicity in Health Care Organisations instrument

**DOI:** 10.5334/ijic.1849

**Published:** 2015-11-23

**Authors:** José Joaquín Mira, Roberto Nuño-Solinís, Paloma Fernández-Cano, Joan Carlos Contel, Mercedes Guilabert-Mora, Olga Solas-Gaspar

**Affiliations:** Alicante-Sant Joan d’Alacant Health District, Consellería de Sanidad, Alicante and Professor, Miguel Hernández University, Elche, Alicante, Spain; Deusto Business School Health, University of Deusto, Bilbao, Spain; Merck Sharp & Dohme España (MSD), Madrid, Spain; Chronic Prevention and Care Programme, Department of Health, Servicio Catalán de la Salud (CatSalut), Barcelona, Spain; Department of Health Psychology, Miguel Hernández University, Elche, Alicante, Spain; International Consultant on Health Policies and Management Organizations and Associated Researcher in New Health Foundation, Spain

**Keywords:** chronic illness, measurement, quality improvement, self-assessment

## Abstract

**Introduction:** The Instrumento de Evaluación de Modelos de Atención ante la Cronicidad/Assessment of Readiness for Chronicity in Health Care Organisations instrument was developed to implement the conceptual framework of the Chronic Care Model in the Spanish national health system. It has been used to assess readiness to tackle chronicity in health care organisations. In this study, we use self-assessments at macro-, meso- and micro-management levels to (a) describe the two-year experience with the Instrumento de Evaluación de Modelos de Atención ante la Cronicidad/Assessment of Readiness for Chronicity in Health Care Organisations tool in Spain and (b) assess the validity and reliability of this instrument.

**Methods:** The results from 55 organisational self-assessments were included and described. In addition to that, the internal consistency, reliability and construct validity of Instrumento de Evaluación de Modelos de Atención ante la Cronicidad/Assessment of Readiness for Chronicity in Health Care Organisations were examined using Cronbach's alpha, the Spearman–Brown coefficient and factorial analysis.

**Results:** The obtained scores reflect opportunities for improvement in all dimensions of the instrument. Cronbach's alpha ranged between 0.90 and 0.95 and the Spearman–Brown coefficient ranged between 0.77 and 0.94. All 27 components converged in a second-order factorial solution that explained 53.8% of the total variance, with factorial saturations for the components of between 0.57 and 0.94.

**Conclusions:** Instrumento de Evaluación de Modelos de Atención ante la Cronicidad/Assessment of Readiness for Chronicity in Health Care Organisations is an instrument that allows health care organisations to perform self-assessments regarding their readiness to tackle chronicity and to identify areas for improvement in chronic care.

## Introduction

The complex needs of people suffering from chronic conditions, particularly those with multimorbidity, highlight the need to develop person-centred integrated care models. Acute-oriented and hospital-centred models are increasingly inefficient and unsustainable for tackling chronicity [1].

The Chronic Care Model [2] and related frameworks [3,4] provide a model for delivering care to chronic patients with evidence-based interventions tested across a wide range of chronic conditions, including multimorbid patients [5–7].

In recent years, in Spain, there have been changes in the way chronic care is being organised together with other initiatives that take the Chronic Care Model [8,9] as a reference. For instance, in the implementation of policies, plans and strategies for chronic diseases at national and regional levels many new interventions and programmes have been developed and deployed, such as nurse case managers, active patient programmes, integrated care pathways, shared electronic medical records, non-face-to-face consultations, etc.

The Chronic Care Model promotes a comprehensive approach in which no single intervention component is the key ingredient for success but rather takes into account the synergic effect of a multi-level intervention approach. Therefore, multidimensional intervention packages that incorporate the different dimensions of the Chronic Care Model seem to be most effective [10,11]. The underlying idea is to transform the current acute-based model for patients with chronic diseases towards a proactive, planned, integrated and population-based care. Although primary care is seen as the main care provider and coordination hub, it is recognised that an appropriate mix of care settings is required within the context of an integrated care system. The Chronic Care Model has been shown to provide benefits for chronically ill patients and its implementation in many countries is increasing [12].

To measure progress within the Chronic Care Model, the Assessment of Chronic Illness Care [13] has been used in many settings, particularly in systems where multiple private insurers and providers play a greater role, and operate mainly in non-monopolistic delivery systems. Studies to measure the psychometric properties of Assessment of Chronic Illness Care have been performed in Germany [14], the Netherlands [15], Thailand [16] and the USA [17]. They have shown that Assessment of Chronic Illness Care has internal consistency and sensitivity. In an effort to adapt Assessment of Chronic Illness Care to the Spanish Health System, it was considered that it requires a contextual adaptation due to the nature of the Spanish health system (tax funded and almost free at the point of use, providing universal care by an oligopoly of public providers); and that further substantial changes were needed due to the following reasons: (a) the need of a population health approach instead of a disease-oriented focus; (b) role of primary care as gatekeeper and main coordinator of chronic care in the Spanish Health System; (c) new and emerging evidence of several interventions for patients with chronic conditions, particularly for multimorbid patients; and (d) the role of proven technological innovations (electronic medical record, remote monitoring, social networks, predictive models, personal health records, etc.).

Therefore, a new instrument called [18] Instrumento de Evaluación de Modelos de Atención ante la Cronicidad/Assessment of Readiness for Chronicity in Health Care Organisations was developed inspired by Assessment of Chronic Illness Care and the tool developed by Pearson et al [19]. Since 2011, Instrumento de Evaluación de Modelos de Atención ante la Cronicidad/Assessment of Readiness for Chronicity in Health Care Organisations has been used in Spain as a self-assessment tool in the context of regional strategies for tackling chronicity.

In this study, we use self-assessments at the macro-, meso- and micro-managerial levels to (a) describe our two-year experience with the Instrumento de Evaluación de Modelos de Atención ante la Cronicidad/Assessment of Readiness for Chronicity in Health Care Organisations instrument in Spain in such contexts and (b) assess the validity and reliability of this instrument.

## Methods

### Sample and settings

A descriptive study was performed based on the analysis of 55 self-assessments undertaken between April 2011 and December 2013. Self-assessments were conducted in four Regional (Autonomous Communities) Health services (macro), 29 health districts or integrated delivery organisations (meso) and 22 primary health care centres (micro) were analysed. These dimensions are structured around the three levels where integration can take place: the macro (system) level, the meso (organisational) level and the micro (clinical) level [20].

### The tool

Instrumento de Evaluación de Modelos de Atención ante la Cronicidad/Assessment of Readiness for Chronicity in Health Care Organisations is an instrument that can be self-administered by health organisations at macro, meso and micro levels. Implementation is assessed by way of a scale that combines deployment, systematic evaluation and orientation to improvement. Instrumento de Evaluación de Modelos de Atención ante la Cronicidad/Assessment of Readiness for Chronicity in Health Care Organisations uses a systemic, population-based approach and integrates the full spectrum of promotion, prevention, care and cure, including coordination with social services. This instrument was structured into a taxonomy of 6 dimensions, 27 components and 80 interventions, along with a 0–100 scale for each intervention. This instrument allowed the self-assessment of health care organisations as regards their degree of readiness to provide integrated care for coping with chronicity. Decision-makers, health managers, a whole range of clinicians, patients and other experts contributed to the design and validation of this instrument. Instrumento de Evaluación de Modelos de Atención ante la Cronicidad/Assessment of Readiness for Chronicity in Health Care Organisations was piloted in six health organisations at different decision levels (macro, meso and micro) [18].

### The self-assessment process

The self-assessment process was facilitated by a team of the authors of the paper. In order to perform the self-assessments, Instrumento de Evaluación de Modelos de Atención ante la Cronicidad/Assessment of Readiness for Chronicity in Health Care Organisations and its assessment scale were explained and later the managerial team of each organisation subsequently agreed on the score for each intervention in accordance with the instructions [21].

Self-assessment sessions with Instrumento de Evaluación de Modelos de Atención ante la Cronicidad/Assessment of Readiness for Chronicity in Health Care Organisations were performed in small groups of professionals, ranging from 5 to 12 in most cases and including the top management profiles (executive director, medical director, nursing director, etc.) plus some clinical leaders who exchanged data, experiences and judgement to reach a consensus rating for each intervention. This approach facilitates analysis and insight into the current status of the care provided by their own organisation to chronic patients as well as the identification and commitment to an own improvement pathway for every organisation or system assessed.

### Validation and refinement

To assure that the structure and contents of the Instrumento de Evaluación de Modelos de Atención ante la Cronicidad/Assessment of Readiness for Chronicity in Health Care Organisations were still the right ones, the deployment team members shared their experiences of conducting self-assessments and the results of the working group sessions were reviewed by consensus. This analysis allowed to identify comprehension difficulties when reporting some of the evaluated interventions and repetitions of the same type of intervention (although with nuances). The elimination or merging of several interventions was the results of consistent comments from members of the assessment teams, who judged them redundant to other interventions.

In addition, the floor and ceiling effects for the scores were assessed, as were the internal consistency (Cronbach's alpha) and the reliability of the split halves using the Spearman–Brown coefficient. Spearman's rho statistic was used to calculate the intercorrelations between the six dimensions.

### Statistical analysis

The results of the self-assessments were analysed on the basis of the scores for each dimension and extreme values for the interventions in each dimension. The self-assessments for the three contexts were compared using the non-parametric Kruskal–Wallis test. The 50th and 70th percentiles were considered as segmentation points to describe the interventions with the highest and lowest degrees of implementation in Spain.

## Results

### Readiness for chronicity in Spain

The mean scores for the Instrumento de Evaluación de Modelos de Atención ante la Cronicidad/Assessment of Readiness for Chronicity in Health Care Organisations dimensions in the micro-context were higher than those in the macro- and meso-contexts, except for the community health dimension (Table 1). The health care model dimension gave the highest score for macro- and meso-organisations as well as at a micro-level. The Organisation of the Health System dimension received the worst assessment for macro-organisations.

Whereas some coincidences were found among the three contexts (macro, meso and micro) for those interventions with limited implementation in Spain, the same cannot be said as regards identification of those interventions that had achieved a higher level of roll-out, as reflected in their higher scores (Table 3). The former includes a delay in implementing technological solutions that allow patients to remotely interact with the health system. In the opinion of the participants in the self-assessment sessions, the interventions with highest roll-out include interventions from the information systems dimension at the micro-level. The highest scores in the meso- and macro-levels coincided with teamwork, patient identification systems and the availability of a reference for the patient at each care level. In contrast to the macro- and meso-contexts, at a micro-level the professionals reported that they received information regarding clinical and management indicators and from prescribing aids (Table 2).

In the macro-level, the highest number of interventions that would require a greater intensity as regards implementation was associated with the system organisation and information systems dimensions (Table 3). These were followed by self-care-related interventions. In the meso-context, the findings showed greater deficiencies in the self-care, decision-making support and information systems dimensions, whereas in the micro-level the self-assessments highlighted the need to concentrate on interventions in the community health, self-care and care model dimensions.

### Validation and refinement of Instrumento de Evaluación de Modelos de Atención ante la Cronicidad/Assessment of Readiness for Chronicity in Health Care Organisations instrument

Experience in the running of self-assessments and consensus among the research team members suggested the elimination or merging of five interventions, namely 1.2.3 (follow-up of chronicity strategic planning), 1.4.2 (output measures, included in 1.2.2, outcome indicators), 3.6.2 (use of telemonitoring, included in 3.6.1, non-face-to-face care), 4.1.2 (participation of caregivers in assessment for patient's self-care, included in the generic 4.1.1 patient's assessment for self-care) and 4.3.2 (providing mutual support, joined in 4.3.3 patient's participation in associations, mutual support groups), as their contents were redundant in light of other interventions as well as minor/very minor modifications in 23 interventions for a greater readability and understanding.

All data were calculated according to the Instrumento de Evaluación de Modelos de Atención ante la Cronicidad/Assessment of Readiness for Chronicity in Health Care Organisations 75 elements (interventions) version resulting from this analysis. Regarding the statistical validation, no floor or ceiling effect was detected for any of the interventions. Cronbach's alpha ranged between 0.90 and 0.95 for the six dimensions studied, and the Spearman–Brown coefficient ranged between 0.77 and 0.94 (Table 4). The mean scores for the dimensions ranged between 26.0 points for community health and 35.3 for information systems (maximum score of 100 points). All 27 components converged in a second-order factorial solution that explained 53.8% of the total variance, with factorial saturations for the components of between 0.57 and 0.94.

## Discussion

Instrumento de Evaluación de Modelos de Atención ante la Cronicidad/Assessment of Readiness for Chronicity in Health Care Organisations is an instrument developed to implement the conceptual framework of the Chronic Care Model in the Spanish national health system. It has been used to assess readiness to tackle chronicity in health care organisations.

Instrumento de Evaluación de Modelos de Atención ante la Cronicidad/Assessment of Readiness for Chronicity in Health Care Organisations was framed within traditional quality improvement theories and methods [19,22], the Chronic Care Model for the included interventions and the European Foundation for Quality Management for the assessment scale. Although organisational readiness is recognised as a potential facilitator of effective knowledge translation, there is currently a lack of consensus regarding how to assess it [23]. New advances in the field of implementation and knowledge translation in chronic care can help to further develop or complement this tool and demonstrate that organisational readiness constitutes an actionable concept in order to assess organisational capacity to engage in implementing change in chronic care [24].

This tool was inspired by Assessment of Chronic Illness Care, which has been shown to demonstrate adequate validity, internal consistency and reliability. The experience of other European countries confirms the need to adapt such self-assessment instruments to the specific health system context [14]. Instrumento de Evaluación de Modelos de Atención ante la Cronicidad/Assessment of Readiness for Chronicity in Health Care Organisations showed a greater consistency than that found when applying Assessment of Chronic Illness Care in Germany [14] and very similar to those found in validation studies in the Netherlands [15] and Thailand [16].

Other major drivers for the adaptation of Assessment of Chronic Illness Care to our context have been the desire to capture the progress on health information and communication technologies since mid-1990s when Assessment of Chronic Illness Care was developed. Technologies as electronic medical and personal records, population risk stratification or telemonitoring have shown their usefulness in improving chronic patient care in different settings and have been incorporated in the new instrument. Besides, Instrumento de Evaluación de Modelos de Atención ante la Cronicidad/Assessment of Readiness for Chronicity in Health Care Organisations follows a comprehensive population health approach from end to end, since community health to the coordination or integration with social services. All this together and its applicability to the three decision levels of health care organisations might signal Instrumento de Evaluación de Modelos de Atención ante la Cronicidad/Assessment of Readiness for Chronicity in Health Care Organisations as a second generation tool for self-assessment of chronic care.

As is the case for the Assessment of Chronic Illness Care dimensions, the scores for the six Instrumento de Evaluación de Modelos de Atención ante la Cronicidad/Assessment of Readiness for Chronicity in Health Care Organisations dimensions are interrelated, thus confirming that the interventions converge on a second-order factorial structure with a single factor with satisfactory saturations. This instrument contains a set of interventions that can be used as a roadmap by decision-makers, managers and clinical leaders interested in building a first-class integrated chronic care model. The Chronic Care Model evidence base shows that its deployment may improve processes and outcomes [22,25]. However, self-assessment scores show the difficulty and slow pace in achieving transformational change, which has also been suggested in other studies [26].

This study shows a consistent gradient in the rating of the six dimensions, which are lower at the macro-level and higher at the micro-level, with the meso-level falling between these two extremes. A likely explanation for these differences might be the type of organisation evaluated. Many of the micro-organisations assessed were primary care, mental health or integrated care services that had a record of several years of efforts to improve chronic care, whereas the macro-organisations were Regional Health Services that wanted to have a baseline diagnosis before starting to implement strategies to tackle chronicity. This might also explain the low rating achieved in the health system organisation dimension by these entities. There may, however, be other possible explanations as the same pattern has been observed in non-formal assessment sessions, thereby possibly suggesting a higher level of exigency among decision-makers or a lack of information about the real progress being made in the centres.

The scores for those interventions which, in general, did not exceed 50 points, reflected the absence of an assessment culture as the measurement scale requires that the interventions implemented were systematically assessed in order for this limit to be exceeded. This drawback, together with the heterogeneity of information systems deployment, is in accordance with the findings of other studies as regards being characteristic of the approaches to chronicity care in Europe [27]. Advances in care integration should be expected to be easier in the Spanish model due to the presence of population-based primary care, yet it lacks the set of attributes you would expect in an integrated model [28].

This study helps to identify those interventions in the Chronic Care Model framework with a lower level of implementation in Spain. In this case, those related to participation of the patient in decision-making or in setting care objectives with the physician are highlighted. How the health system itself promotes participation of the patient in fora, associations or self-help groups also stands out. There is a need to make further progress in developing information systems that favour greater integration between care levels and also that allow professionals to rely on support systems, for example, alarms in the event of inadequate patient control or when the patient is transitioned between care levels or settings. Experiences that link professionals’ incentives to indicators reflecting good patient management remain undeveloped. The perception found at the micro-level concerning the development of programmes at a community level remains fragile and is therefore an area requiring intervention in the coming years.

### Limitations

The theoretical background of Instrumento de Evaluación de Modelos de Atención ante la Cronicidad/Assessment of Readiness for Chronicity in Health Care Organisations is based on quality improvement, recent theoretical advances have not been fully included in the tool [29], nor the latest evidence in implementation of chronic care models [30].

The presented self-assessment results reflect the opinion of all those who participated in the self-assessment sessions. The sub-sample for the macro-context is reduced to four regional health services. There is a selection bias that prevents the results from being generalised to Spain as a whole, as the people responsible for carrying out the self-assessment have designed strategies and made further progress in integrated care with respect to other parts of the country. This bias results from the fact that self-assessments were carried out in those regions and organisations most concerned and experienced in fostering a care model change.

The macro-, meso- and micro-self-assessments do not correspond to a single Autonomous Community, therefore meaningful comparisons cannot be drawn between clinical and management viewpoints. As the self-assessments did not include population-based indicators or those based on the patients’ perception of their experience or on efficiency, they simply reflect the opinion of all those who participated in them. The Instrumento de Evaluación de Modelos de Atención ante la Cronicidad/Assessment of Readiness for Chronicity in Health Care Organisations scores do not allow results to be evaluated in terms of health gain indicators. However, these findings constitute the highest number of self-assessments in Spain for tackling chronicity using the same assessment approach and same instrument.

Future studies should evaluate the solidity of this self-assessment instrument over time, check whether the implementation of these interventions has a positive effect on the clinical results, perception of care and efficiency, identify those interventions in which the highest improvement increases in terms of patient care and results are achieved over time and determine whether the interventions proposed continue to serve the purpose of guiding decision-making regarding which actions (interventions in Instrumento de Evaluación de Modelos de Atención ante la Cronicidad/Assessment of Readiness for Chronicity in Health Care Organisations) can be introduced with some degree of confidence concerning their utility.

## Conclusions

Instrumento de Evaluación de Modelos de Atención ante la Cronicidad/Assessment of Readiness for Chronicity in Health Care Organisations is an instrument that allows the self-assessment of organisational readiness to tackle chronicity and helps to identify areas for improvement that will allow further progress to provide better care for chronic patients. Identification of these areas for improvement promotes a more “systemic”, wide-ranging and multidimensional approach as a strategy for progress, recognising the efforts and synergies arising from the combination of Chronic Care Model interventions. These findings suggest the need to make further progress in new ways of fostering patient participation, taking advantage of communication technologies to encourage innovation or to favour a newer non-face-to-face care model. Moreover, they confirm the need of more systematic evaluation of interventions. Finally, further development of current information systems must favour integration between care levels, which remains an objective that still requires much greater efforts at all managerial levels.

## Figures and Tables

**Table 1.**
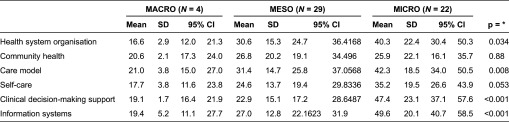
Scores for IEMAC/ARCHO dimensions in a macro, and micro context

*Difference of means obtained using the Kruskal-Wallis test for non-parametric independent samples.

**Table 2.**
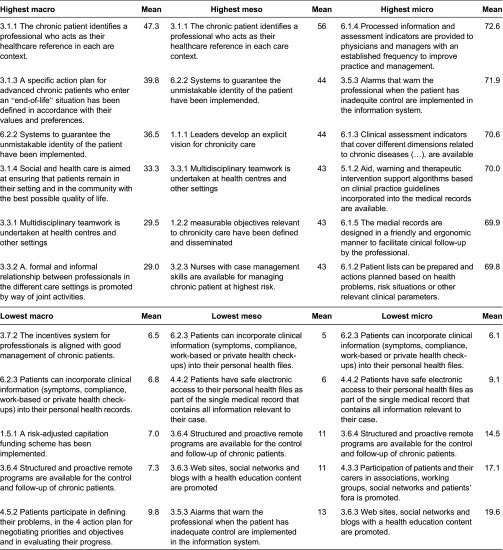
Interventions with the highest and lowest scores in a macro and micro context

**Table 3.**
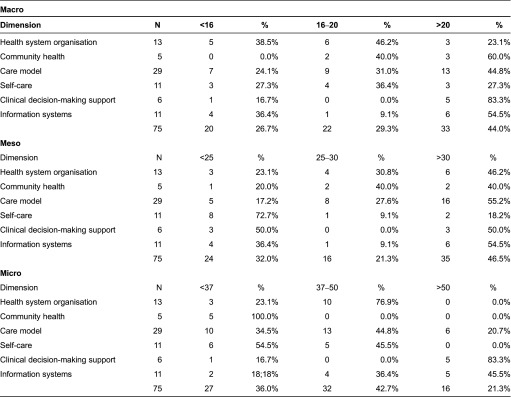
Distribution of the scores for IEMAC/ARCHO interventions in a macro, and micro context

**Table 4.**
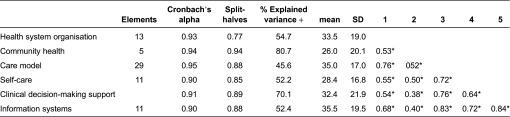
Internal consistency, reliability and intercorrelations between IEMAC/ARCHO dimensions

*Result of the principal components factor analysis.

## Notes

Competing interests: Instrumento de Evaluación de Modelos de Atención ante la Cronicidad/Assessment of Readiness for Chronicity in Health Care Organisations has been developed by RN, PF, JJM, JCC, MG and OS. It is property of the University Miguel Hernández, the Basque Institute for Health Care Innovation and MSD. It is provided free for non-profit organisations that want to improve chronic patient care. PF works at Merck Sharp & Dohme Spain. This study has been funded by Merck Sharp & Dohme Spain.

Authors’ contributions: JJM, RN and MG conceived the structure of this article. PF, MG, OS and JJM conducted the sessions of self-assessment with Instrumento de Evaluación de Modelos de Atención ante la Cronicidad/Assessment of Readiness for Chronicity in Health Care Organisations. RN and JJC revised the European experiences using Chronic Care Model. JJM, MG and OS performed statistical analysis. JJM, MG, PF and OS prepared a first version of this original. All authors read and approved the final manuscript.
